# Interactions between *Blastocystis* subtype ST4 and gut microbiota in vitro

**DOI:** 10.1186/s13071-022-05194-x

**Published:** 2022-03-08

**Authors:** Lei Deng, Kevin S. W. Tan

**Affiliations:** Laboratory of Molecular and Cellular Parasitology, Department of Microbiology and Immunology, Healthy Longevity Translational Research Programme, Yong Loo Lin School of Medicine, National University of Singapore, 5 Science Drive 2, Singapore, 117545 Singapore

**Keywords:** *Blastocystis*, Gut microbiota, ROS, Epithelial barrier, Co-incubation

## Abstract

**Background:**

*Blastocystis* ST4 is a common protistan parasite of the gastrointestinal tract of humans and a wide range of animals. While it has been suggested that colonization with ST4 is associated with healthy gut microbiota, how ST4 influences the gut microbiota remains poorly studied. This study aimed to examine the interactions between ST4 and several intestinal bacteria using in vitro co-culture systems, and to further investigate the mechanism of interaction and its effect on the epithelial barrier integrity of HT-29 cells.

**Methods:**

Seven intestinal bacteria *Bacteroides fragilis*, *Bifidobacterium longum*, *Bacillus subtilis*, *Bacteroides vulgatus*, *Escherichia coli*, *Enterococcus faecalis*, and *Lactobacillus brevis* were co-cultured with *Blastocystis* ST4 in vitro. Flow cytometry and quantitative reverse-transcription polymerase chain reaction (qRT-PCR) were used to determine the role of reactive oxygen species (ROS) and bacteria oxidoreductase genes, respectively, in response to *Blastocystis* co-incubation. Transepithelial electrical resistance (TEER) and flux assays were performed to assess the effect of microbiota representatives on the integrity of the intestinal epithelial barrier.

**Results:**

Co-incubation with *Blastocystis* ST4 showed a beneficial influence on most intestinal bacteria, while ST4 significantly inhibited the growth of *B. vulgatus*, a common pathogen in the genus *Bacteroides*. The decrease in *B. vulgatus* when co-incubated with *Blastocystis* ST4 was associated with high levels of ROS and the upregulation of oxidative stress-related genes. Furthermore, co-incubation with *Blastocystis* ST4 was able to protect the intestinal epithelial barrier from damage by *B. vulgatus*.

**Conclusions:**

This study demonstrated, for the first time, that *Blastocystis* ST4 has beneficial effects on intestinal commensal bacteria in vitro, and can inhibit the growth of pathogenic *B. vulgatus*. Combined with previous microbiome research on ST4, our data suggest that ST4 may be a beneficial commensal.

**Graphical Abstract:**

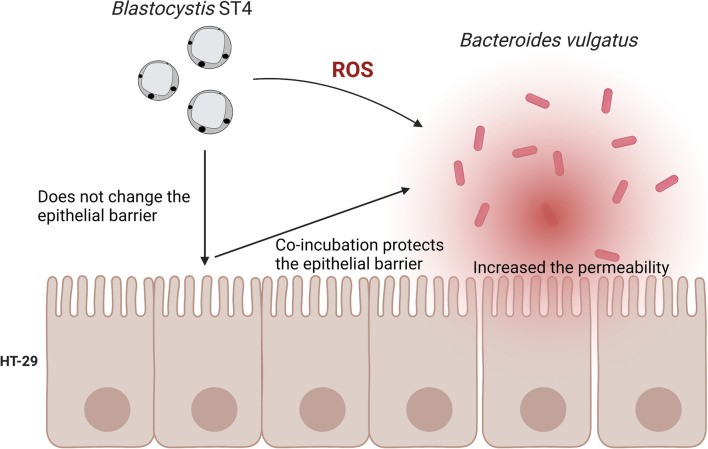

**Supplementary Information:**

The online version contains supplementary material available at 10.1186/s13071-022-05194-x.

## Background

*Blastocystis* is a common single-celled intestinal eukaryote that colonizes the gastrointestinal tract of humans and animals. There are an estimated more than 1–2 billion people colonized with *Blastocystis* worldwide [1]. The clinical significance of *Blastocystis* is still unclear, although it has been widely studied for more than 100 years [2]. Recent studies reported *Blastocystis* infection decreases the proportion of beneficial bacterial, such as *Bifidobacterium* and *Lactobacillus* [3, 4]. However, the majority of individuals colonized with *Blastocystis* are asymptomatic and harbor higher gut bacterial diversity and healthy gut microbiota [5, 6]. These discrepancies may be influenced by the complex nature of *Blastocystis* wherein several genetically distinct subtypes exist [7].

To date, 25 subtypes have been identified in humans and a wide range of animals based on the analysis of small subunit ribosomal RNA (SSU rRNA) gene [8]. Among them, ST1-4 are the predominant subtypes in humans, accounting for more than 90% of human cases [9]. The prevalence of *Blastocystis* ST4 seems to be influenced by geography, as it is mainly reported in Europe and rarely found in South America, Africa, and Asia [10, 11]. There are only two studies that report on relationships between *Blastocystis* ST4 and gut microbiota. ST4 colonization was positively associated with the abundance of *Sporolactobacillus* and *Candidatus* Carsonella in Swedish travelers [12]. In addition, a higher proportion of beneficial bacteria *Akkermansia* was observed in Flemish healthy individuals colonized with *Blastocystis* ST4 [6].

Nourrisson et al. [13] reported that the proportion of *Bifidobacterium* spp. was decreased in patients with irritable bowel syndrome (IBS) who were colonized with *Blastocystis*, and lower abundance of *Faecalibacterium prausnitzii* was observed in healthy *Blastocystis*-positive individuals, suggesting that *Blastocystis* may be used as an indicator of microbiota changes associated with intestinal dysbiosis. Nevertheless, Terveer et al. included *Blastocystis*-positive (ST1 and ST3) donor samples for fecal microbiota transplantation (FMT) treatment of recurrent *Clostridium difficile* infection (rCDI), and as a result demonstrated that the presence of *Blastocystis* ST1 and ST3 from donors did not cause any adverse gastrointestinal symptoms or any significant effect on the treatment outcome [14]. However, it is not clear how *Blastocystis* affects the gut microbial community.

In the current study, we focused on *Blastocystis* ST4, the most common subtype in the Flemish Gut Flora Project (FGFP) [6], TwinsUK [15], and the American Gut Project (AGP) [16]. We investigated, in vitro, the interactions between *Blastocystis* ST4 and representative gut microbiota bacteria. Our findings provide valuable insights into the mechanism by which *Blastocystis* affects the gut microbiota.

## Methods

### Culture of *Blastocystis*

Two axenized *Blastocystis* isolates (WR1 and WR2) belonging to subtype 4 were used in this study. Both isolate WR1 and WR2 were originally isolated from healthy Wistar rats during an animal survey at the National University of Singapore (NUS) [17]. *Blastocystis* ST4 was maintained in 10 ml of pre-reduced Iscove’s modified Dulbecco’s medium (IMDM) (Gibco) supplemented with heat-inactivated 10% horse serum (Gibco). Cultures were incubated under anaerobic conditions in an AnaeroJar (Oxoid) with gas pack (Oxoid) at 37 °C and subcultured every 3–4 days. *Blastocystis* cell counts were done manually using a hemocytometer (Kova International).

### Bacterial strains

A lyophilized stock of the intestinal representative bacteria was obtained from the American Type Culture Collection (ATCC, Rockville, MD, USA). The commensal bacteria *Escherichia coli* ATCC 11775, *Enterococcus faecalis* ATCC 29212, and *Bacillus subtilis* ATCC 6633 were cultured and maintained in Luria–Bertani (LB) broth and agar (Sigma). *Bifidobacterium longum* ATCC 15707 was grown and maintained in Bifidus selective medium (BSM) broth and agar (Sigma). *Lactobacillus brevis* ATCC 14869 was cultivated on deMan, Rogosa and Sharpe (MRS) medium (Sigma). The microorganisms causing opportunistic infections *Bacteroides fragilis* ATCC 25285 and *Bacteroides vulgatus* ATCC 8482 were grown in brain heart infusion (BHI) broth (Sigma), and the number of colony-forming units (CFU) of *Bacteroides* was counted on Trypticase soy agar with defibrinated sheep blood (BD). All the bacteria were cultured at 37 °C. *Bacteroides fragilis*, *B. longum*, and *B. vulgatus* were incubated in a jar equipped with Anaerogen gas packs (Oxoid).

### Co-culture experiments

The protocol for co-culture of *Blastocystis* and bacteria was described in our previous publication [3]. Briefly, *Blastocystis* and bacteria were washed three times with pre-reduced sterile phosphate-buffered saline (PBS), respectively. Next, 1 × 10^9^ CFU/ml of each bacteria strain was added to 1 × 10^7^ cells/ml of *Blastocystis*, which were suspended in 1 ml pre-reduced PBS. Controls consisted of only 1 × 10^7^ cells/ml of *Blastocystis* and only 1 × 10^9^ CFU/ml of bacteria suspended in 1 ml pre-reduced PBS. After incubation for 24 h at 37 °C, the number of *Blastocystis* cells was determined using a hemocytometer, and the drop plate method was used to determine the number of bacteria CFU. All co-culture experiments were performed in triplicate and repeated at least three times.

### ROS staining and flow cytometry

To determine the cellular reactive oxygen species (ROS) content of *B. vulgatus* after co-culture, BacLight Red (Thermo Fisher) and 2′,7′-dichlorofluorescein diacetate (DCFDA) (Sigma) were used. Specifically, *B. vulgatus* were stained with BacLight Red at a concentration of 1 μM for 15 min at room temperature before the co-culture experiment, and then the cells were washed twice using pre-reduced PBS at 1000×*g* for 10 min. After co-culture for 24 h at 37 °C, cells were stained with DCFDA at a concentration of 20 μM for 30 min at 37 °C, and then washed twice in PBS. The cells were run in an Attune Flow Cytometer (Life Technologies) using blue (488 nm) and yellow (561 nm) lasers. Data were analyzed using FlowJo software.

### Gene expression analysis using quantitative reverse-transcription polymerase chain reaction (qRT-PCR)

*Bacteroides vulgatus* mRNA was extracted using the RNeasy Mini Kit (Qiagen, Germany) according to the manufacturer’s instructions. Complementary DNA (cDNA) was synthesized using the iScript cDNA kit (Bio-Rad). All qPCRs were performed with SsoAdvanced™ Universal SYBR Green Supermix (Bio-Rad) on an Applied Biosystems 7500 Fast Real-Time PCR System (Applied Biosystems). qPCR was carried out in a total volume of 20 μl, which consisted of the master mixture and 2 μl of cDNA template. The former contained 10 μl SsoAdvanced™ Universal SYBR Green Supermix (2×), 0.3 mM of each primer, made up to 18 μl with nuclease-free water. The qPCR protocol consisted of denaturing at 95 °C for 10 min, followed by 40 cycles of 95 °C (15 s) and 60 °C (1 min), and the melt curve stage using the instrument default setting. All primers used in the present study are listed (Additional file 1: Table S1).

### Culture of HT-29 cell line

The HT-29 cell line is similar to small intestine enterocytes regarding structure and differentiation process, which can better mimic real responses in vivo [18]. HT-29 stock cultures were maintained in T-75 flasks in a humidified incubator with 5% CO_2_ at 37 °C. Cell cultures were grown in complete Dulbecco’s modified Eagle’s medium (DMEM), consisting of 10% heat-inactivated fetal bovine serum (FBS) (Gibco) and 1% each of sodium pyruvate (Gibco), non-essential amino acids (Gibco), and penicillin–streptomycin (Gibco). Culture health was evaluated using the trypan blue assay and only cultures with > 95% viability were used for the experiments. For transepithelial electrical resistance (TEER) and permeability experiments, cells were grown on Millipore transwell filters with polyester (PET) membranes of 3 μm pore size placed in 24-well tissue culture plates in DMEM with 5% CO_2_ at 37 °C. In order to synchronize cells before experiments, all cultures were serum-starved overnight in antibiotic-free and serum-free DMEM.

### Epithelial resistance

TEER across HT-29 monolayer was measured using Millipore ERS-2 voltohmmeter. HT-29 monolayers were grown on a Millipore transwell system until confluence was reached, and the monolayers were then stimulated for 48 h with 3 mM sodium butyrate in serum-free medium (Sigma-Aldrich). Differentiated HT-29 monolayers were conditioned by 1 × 10^6^ cells/ml *Blastocystis* and 1 × 10^9^ CFU/ml *B. vulgatus* in serum-free DMEM for 24 h at 37 °C in anaerobic conditions.

### FITC-dextran permeability assay

The flux assay was performed using fluorescein isothiocyanate-conjugated Dextran 4000 (FITC-Dextran) (Sigma). After TEER measurement, the epithelial and basolateral compartments were washed twice with Hank’s balanced salt solution (HBSS) (Thermo Fisher). Next, 400 μl of warm HBSS was added to the basolateral compartments, and 200 μl of 100 μg/ml FITC-Dextran was added to the apical compartments. After incubation at 37 °C for 1 h, 300 μl of HBSS containing the Dextran-FITC at the basolateral compartment was collected and transferred to a black 96-well plate (Nunc). Fluorescence was measured using a Tecan Infinite F200 microplate reader at excitation and emission wavelengths of 492 nm and 518 nm, respectively.

### Statistical analysis

Statistical analysis was performed using GraphPad Prism 7 software (GraphPad Software, CA, USA). The unpaired two-tailed Student’s *t*-test was used to evaluate differences between the two groups. One-way analysis of variance (ANOVA) was used to evaluate experiments involving multiple groups. Graphs show mean ± SEM. * *P* < 0.05, ** *P* < 0.01, *** *P* < 0.001.

## Results

### Intestinal bacteria have a positive growth effect on the number of *Blastocystis* ST4 cells in vitro

**Fig. 1:**
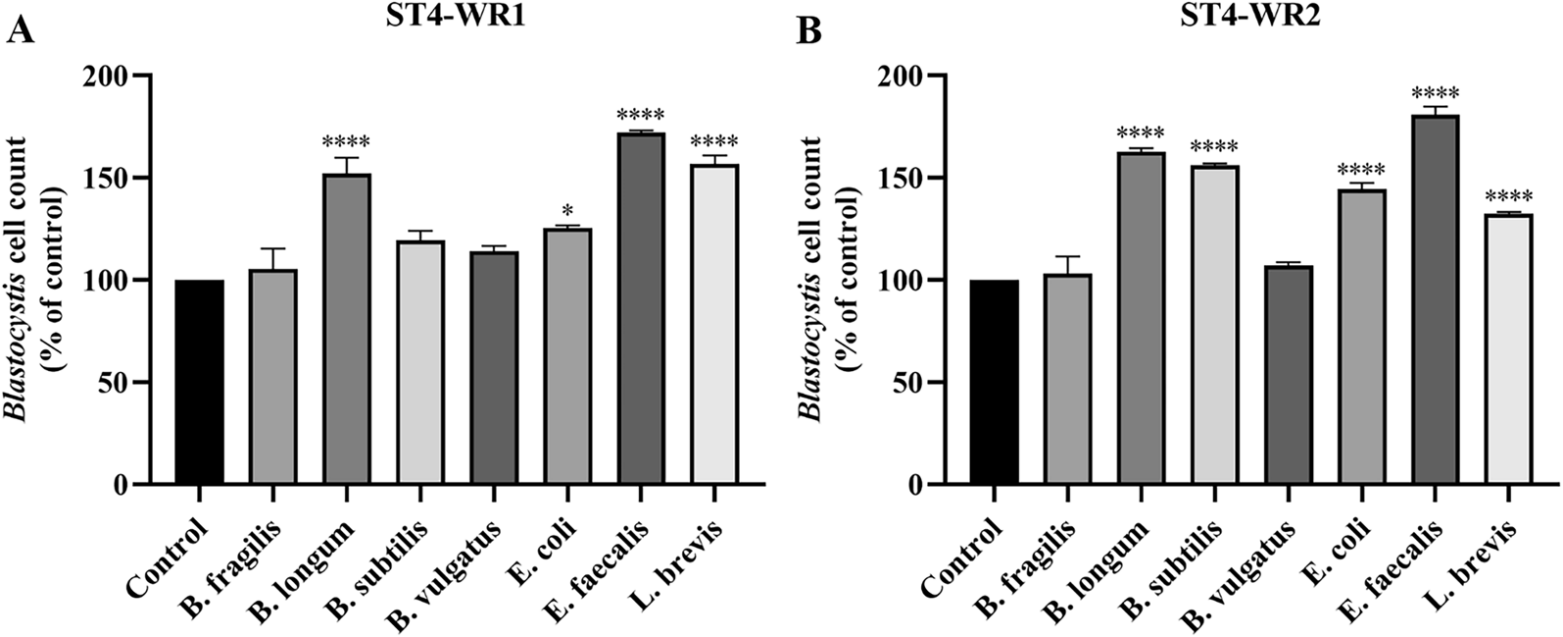
Intestinal bacteria have a positive effect on *Blastocystis* cell count in vitro. 10^7^ cells of *Blastocystis* ST4 were co-cultured with 10^9^ CFU of gut bacteria (*B. fragilis*, *B. longum*, *B. subtilis*, *B. vulgatus*, *E. coli*, *E. faecalis*, and *L. brevis*) in 1 ml of pre-reduced PBS for 24 h at 37 °C. **a** *Blastocystis* ST4-WR1 cell count. **b** *Blastocystis* ST4-WR2 cell count

In order to study the interactions between *Blastocystis* and gut commensal bacteria in vitro, a co-culture system was established as reported previously [3]. This study used two isolates of *Blastocystis* ST4, WR1 and WR2, which were individually co-cultured with representative intestinal bacteria (*B. fragilis*, *B. longum*, *B. subtilis*, *B. vulgatus*, *E. coli*, *E. faecalis*, and *L. brevis*). The number of *Blastocystis* (WR1 and WR2) cells increased significantly when co-incubated with *B. longum*, *E. coli*, *E. faecalis*, and *L. brevis* (Fig. 1a, b). The positive effects were also observed when ST4-WR2 was co-incubated with *B. subtilis* (Fig. 1b). The effects of *B. fragilis* and *B. vulgatus* on the growth of *Blastocystis* WR1 and WR2 were comparable to the *Blastocystis*-only cultures (Fig. 1a, b).

### *Blastocystis* ST4 exerts a positive growth effect on some intestinal bacteria in vitro

**Fig. 2:**
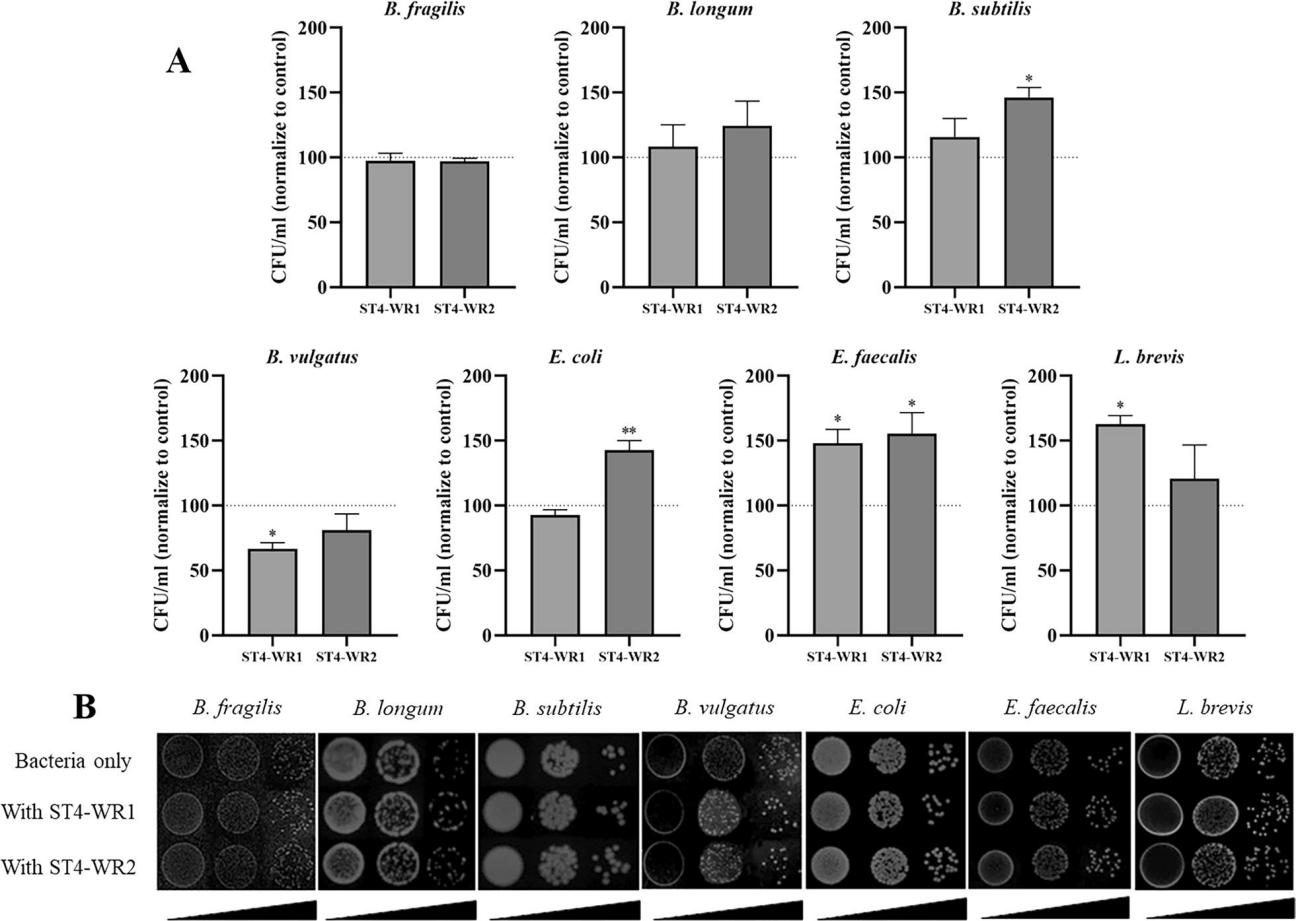
*Blastocystis* ST4 has a positive effect on some intestinal bacteria in vitro. **a** Most gut bacteria co-cultured with *Blastocystis* ST4 showed higher CFU count, while the CFU of *B. vulgatus* was significantly decreased in the presence of *Blastocystis* ST4-WR1. **b** Representative plates of colony-forming units (CFU)/ml

The CFU count of intestinal representative bacteria was determined after incubation with ST4-WR1 or ST4-WR2. Generally, most bacteria had a higher CFU count when co-cultured with ST4-WR1 and ST4-WR2. *Enterococcus faecalis* had a significantly higher CFU count when co-incubated with both ST4-WR1 and ST4-WR2 (Fig. 2a). The CFU of *L. brevis* increased significantly after co-incubation with ST4-WR1 (Fig. 2a). *Bacillus subtilis* and *E. coli* had significantly higher CFU count when co-incubated with ST4-WR2 (Fig. 2a). Interestingly, *B. vulgatus* had a lower CFU count when co-cultured with both ST4-WR1 and ST4-WR2, and a significant difference was observed in ST4-WR1 (Fig. 2a). Representative images of the bacterial colonies on agar plates from the co-incubation assay are shown in Fig. 2b.

### *Blastocystis* ST4 induces oxidative stress on *B. vulgatus *in vitro

**Fig. 3:**
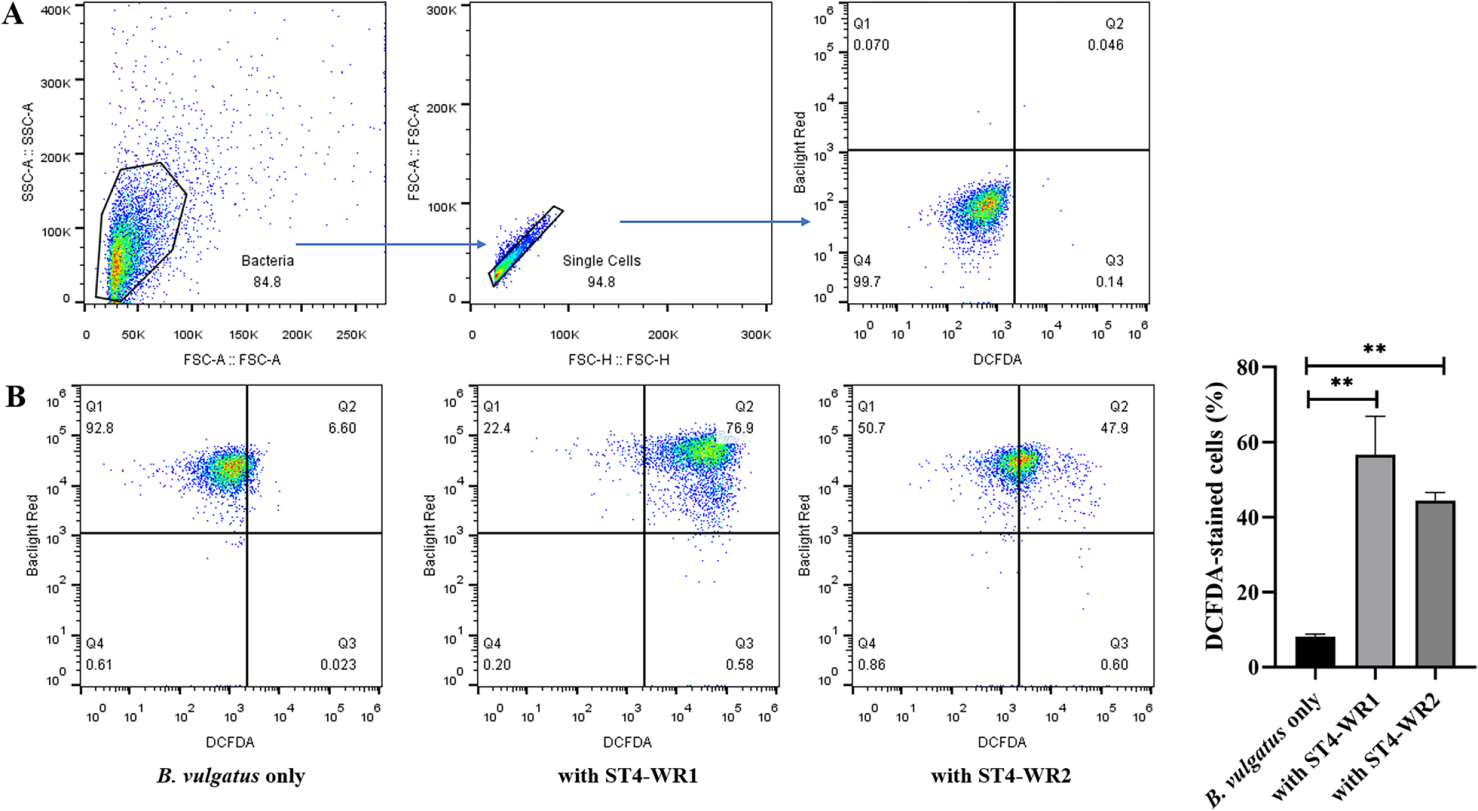
*Blastocystis* induces oxidative stress to *B. vulgatus *in vitro. **a** ROS gating strategies. **b** The representative flow cytometry plots of DCFDA staining. Co-incubation of *B. vulgatus* with *Blastocystis* ST4 displayed significant higher DCFDA content indicating the presence of increased cellular ROS

**Fig. 4:**
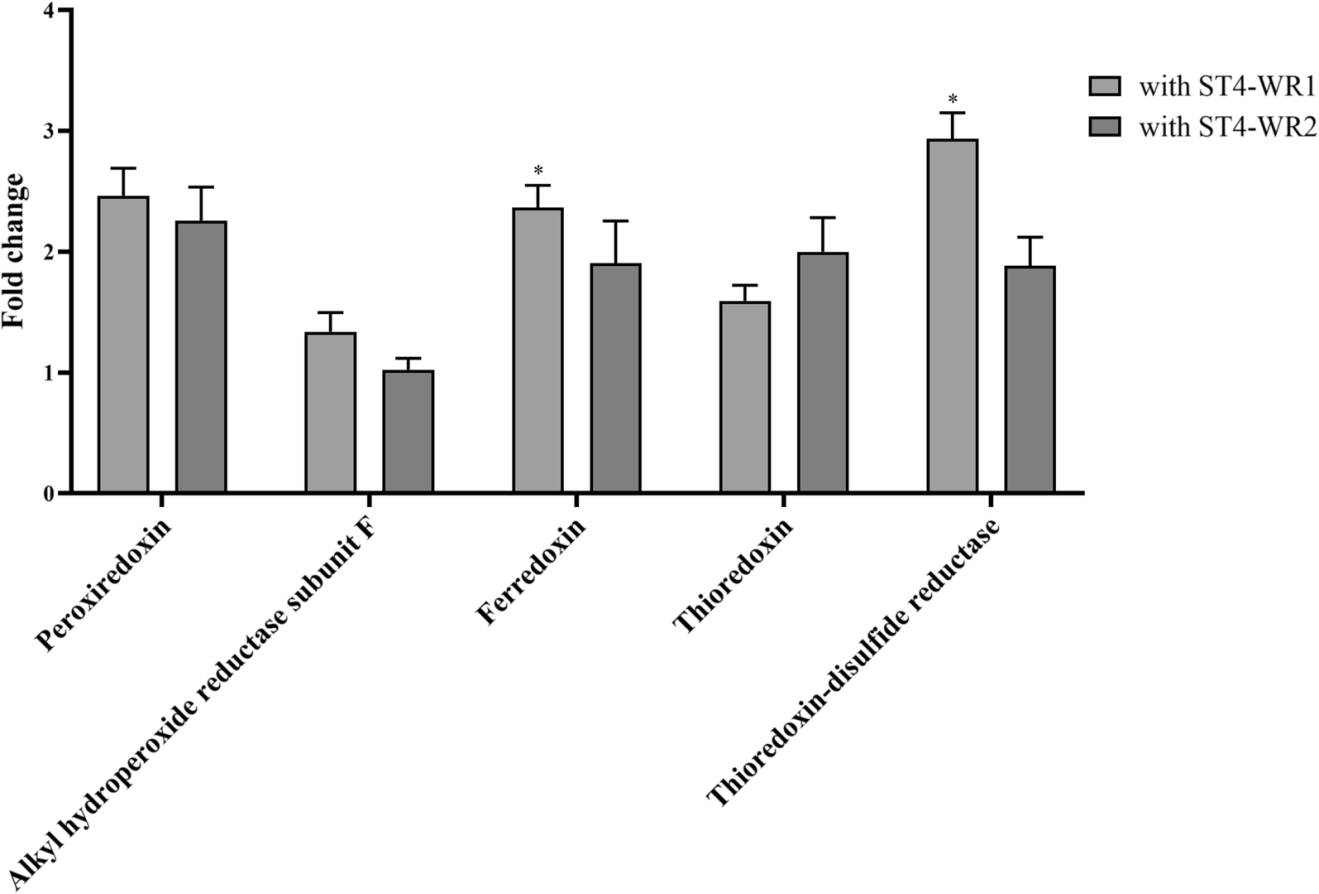
Oxidoreductase gene expression of *B. vulgatus* when incubated with *Blastocystis* ST4-WR1 and ST4-WR2

Oxidative stress refers to the imbalance between the production of ROS and the antioxidant capacity of cells [19], which is one of the typical factors that exacerbate shifts of the microbiota thereby yielding dysbiosis [20]. We further investigated whether *Blastocystis* ST4 affects the viability of *B. vulgatus* through such a mechanism. We used flow cytometry to detect the ROS content in *B. vulgatus* (Fig. 3a). Flow cytometry analysis of DCFDA content showed that co-culture with both ST4-WR1 and ST4-WR2 induced a significant increase in DCFDA content in *B. vulgatus*, indicating the presence of increased cellular ROS (Fig. 3b). In addition, several important genes in response to oxidative stress of *B. vulgatus* were also analyzed including *trxB* (thioredoxin-disulfide reductase), *trxA* (thioredoxin), *ahpC* (peroxiredoxin), *ahpF* (alkyl hydroperoxide reductase subunit F), and *BVU_RS16335* (ferredoxin). The PCR primers used are specific to *B. vulgatus* and do not amplify control *Blastocystis* cDNA (results not shown). Results showed that two of the oxidoreductase genes, *trxB* and *BVU_RS16335*, were upregulated when co-cultured with ST4-WR1 (Fig. 4). These results indicate that the decrease of *B. vulgatus* when co-incubated with *Blastocystis* ST4 was associated with the high content of ROS and the upregulation of oxidative stress-related genes.

### *Blastocystis* ST4 protects intestinal epithelial barrier in vitro

**Fig. 5:**
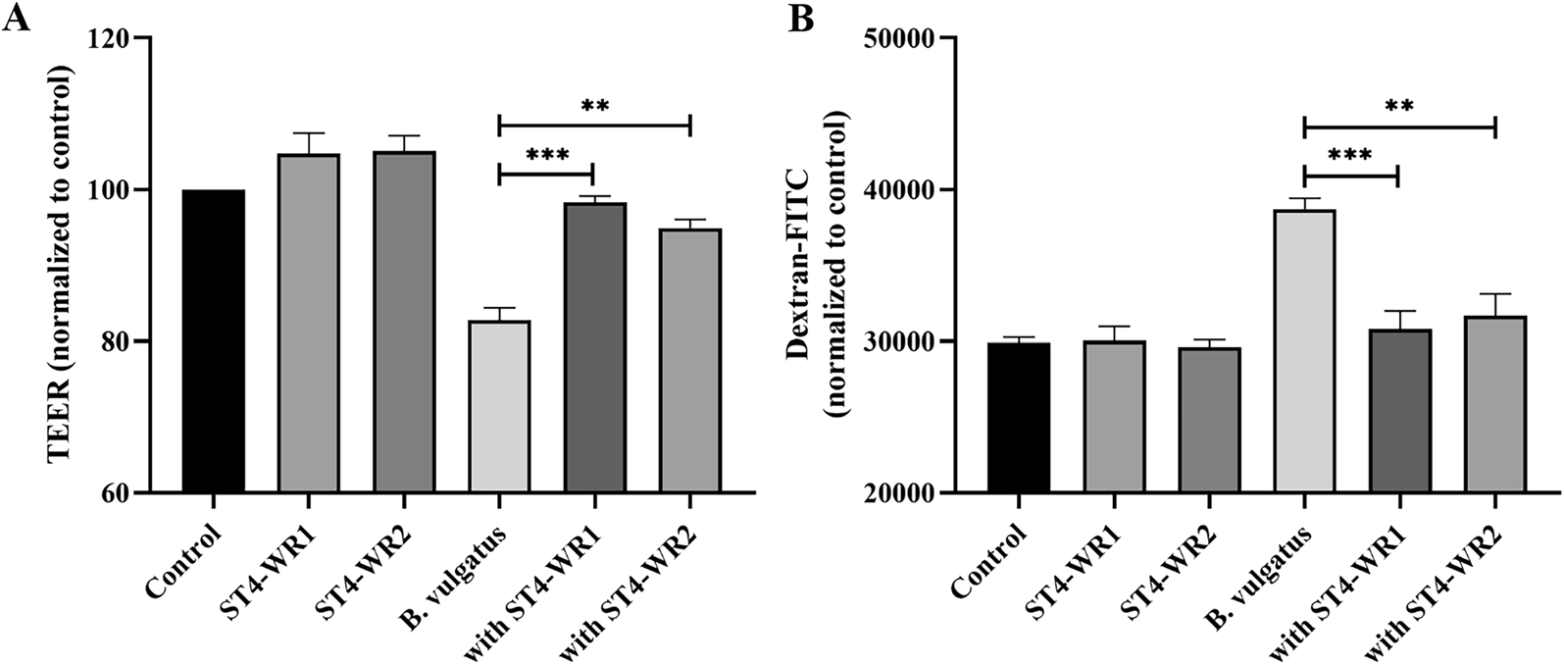
*Bacteroides vulgatus* disrupts the integrity of the epithelial barrier, while co-incubation with *Blastocystis* ST4 prevented the disruption of epithelial integrity (**a**, **b**)

The intestinal barrier protects intestinal cells from opportunistic pathogens in the lumen, which, when compromised, plays a key role in the development of inflammatory bowel diseases [21]. We explored the effect of *Blastocystis* ST4 and *B. vulgatus* on the intestinal epithelial barrier in vitro. ST4-WR1 and ST4-WR2 were individually incubated with *B. vulgatus* apically onto HT-29 cell monolayers differentiated on transwell inserts. TEER measurements showed that neither ST4-WR1 nor ST4-WR2 changed the epithelial barrier significantly compared with that of the control, which is consistent with our previous study on Caco-2 cells [22]. In contrast, *B. vulgatus* exhibited a significant drop in epithelial resistance compared to the control. Interestingly, the epithelial resistance increased significantly when ST4-WR1 and ST4-WR2 were co-cultured with *B. vulgatus* (Fig. 5a). In order to confirm the ST4-mediated protection of the epithelial barrier suggested by an increase in TEER, the flux assay was performed by measuring the flux of FITC across the intestinal epithelial barrier from the apical to the basolateral compartment. As expected, a significant increase in epithelial permeability was observed upon exposure to *B. vulgatus* (Fig. 5b), while the number of reporter molecules decreased significantly when co-cultured with ST4-WR1 and ST4-WR2. Altogether, both assays suggested that *Blastocystis* ST4 can reduce the damage mediated by *B. vulgatus* on the permeability of epithelial cells in vitro.

## Discussion

Although *Blastocystis* has been proposed to be a causative agent of gastrointestinal and dermatological infections, its pathogenic potential and clinical significance remain unclear. *Blastocystis* ST7 decreases beneficial gut bacteria, leading to a dysbiotic state, and was the predominant subtype in diarrheal patients with *C. difficile* infection [3, 23], while the majority of microbiome studies report that *Blastocystis* is associated with higher bacterial diversity and can colonize the human gut for a long time without causing symptoms [5, 6, 10, 24]. Previous reports have reported on the mutualistic relationship between *Blastocystis* and gut commensal bacteria in vitro [3, 25]. The number of *Blastocystis* (ST3 and ST7) cells was higher in the presence of intestinal representative bacteria than in *Blastocystis*-only culture [3, 25]. Here we reported a consistent result in that both ST4-WR1 and ST4-WR2 showed higher *Blastocystis* cells count when co-incubated with intestinal bacteria. The bacteria may produce secretory molecules or metabolites as a nutrient source for *Blastocystis* proliferation.

In this study, *B. fragilis*, *B. longum*, *B. subtilis*, *B. vulgatus*, *E. coli*, *E. faecalis*, and *L. brevis* were selected for the co-incubation assay as representative species of the gut microbiota [26–29]. Among these, *L. brevis* and *B. longum* are well-known probiotic species that have anti-inflammatory properties [30, 31]. Lim et al. reported that the mixture of *B. longum* CH57 and *L. brevis* CH23 ameliorated colitis in mice by inhibiting macrophage activation and restoring the Th17/Treg balance [32]. *Escherichia coli*, *E. faecalis*, *B. subtilis*, and *B. fragilis* are important commensal gut bacteria, which play an important role in human nutrition and health by promoting carbohydrate metabolism, preventing pathogen colonization, and maintaining intestinal immune homeostasis [33]. It should be noted that *B. fragilis* is only a minor component of normal fecal flora, but is more often isolated during infection than the species representing a higher percentage of normal fecal isolates, such as *B. vulgatus* and *B. thetaiotaomicron*, that can be considered as commensals [34]. *Bacteroides vulgatus*, a Gram-negative obligate anaerobe, is linked to higher levels of intestinal inflammation and can cause colitis in mouse models and humans [35–37]. Notably, we mostly observed higher bacterial CFU counts when gut commensal bacteria were co-incubated with *Blastocystis*. The higher bacterial CFU count may be due to bacteria breaking down dead cells from *Blastocystis* or existing bacterial cells to obtain nutrients during *Blastocystis* bacteria co-incubation.

Nonetheless, a significant decrease in CFU count was observed in *B. vulgatus* after co-incubation with *Blastocystis* ST4-WR1. We further explored the underlying mechanism of *Blastocystis* inhibiting *B. vulgatus*, and our results suggested the presence of *Blastocystis* can cause oxidative stress on *B. vulgatus*. Indeed, oxidative stress can shift the microbial compositions by modulating the growth of specific bacterial taxa in the gut, especially exerting antimicrobial activity on strictly anaerobic bacteria [20]. Moreover, ROS can promote pathogen elimination by direct oxidative damage or by a variety of innate and adaptive mechanisms [38]. Defects in the pathogen’s antioxidant mechanism can convert highly virulent pathogens into ROS-sensitive pathogens, indicating that ROS directly damage microorganisms [39]. Additionally, the oxidative damage caused by ROS, such as lipid peroxidation, DNA strand breakage, base oxidation and deamination, and oxidation of methionine residues, can be directly tracked in microorganisms exposed to respiratory burst [40]. However, further clarification on the mechanism of *Blastocystis* causing high ROS in *B. vulgatus* is needed, and other mechanisms of *Blastocystis*-mediated decrease in *B. vulgatus* numbers need to be considered, such as whether pH changes caused by *Blastocystis* culture affect bacterial growth.

The barrier formed by the intestinal epithelium acts as the body’s first line preventing the entry of luminal opportunistic pathogens. Damage to epithelial barrier integrity is commonly associated with intestinal inflammatory diseases [41]. *Bacteroides vulgatus* can produce mucin-degrading enzymes such as glycosidase, sialidases, and neuraminidase, which can profoundly weaken the mucosal barrier function and exaggerate inflammation [42–44]. In vitro experiments demonstrated that *B. vulgatus* can invade colonic epithelial cells (SW-480 and HT-29) and activate the expression of pro-inflammatory cytokines [45]. In contrast, a recent study showed that *B. vulgatus* isolates could attenuate lipopolysaccharide (LPS)-induced IL-8 release from the HT-29 cell line [46], suggesting that different isolates of *B. vulgatus* have different pathological effects on colonic epithelial cells. It has also been determined that the expansion of *B. vulgatus* induces inflammatory gene expression and goblet cell dysfunction in nucleotide-binding oligomerization domain (NOD)-like receptor 2 (*NOD2*) knockout mice [35]. A recent study reported that the level of *B. vulgatus* is linked to higher levels of inflammation and microbial translocation during ART-suppressed HIV infection [47]. Interestingly, in vitro experiments revealed that *Bifidobacterium* (*B. infantis* 1222 and *B. longum* 7052) can suppress the growth of *B. vulgatus* after co-culture [48]. Our study showed, similarly, that co-incubation with *Blastocystis* ST4 can inhibit the ability of *B. vulgatus* to compromise the intestinal epithelial barrier, suggesting *Blastocystis* may play a similar probiotic role in vivo. Future studies should explore whether *Blastocystis* ST4 has similar beneficial effects on the gut microbiota in experimental mouse models and humans.

*Blastocystis* has enormous inter- or intra-genetic variation among subtypes, with different subtypes exhibiting distinct influences on gut microbiota and host immune responses [7, 8, 49]. For example, five strains of *Blastocystis* ST7 (B, C, E, G, and H) showed significant variations in cysteine protease activity, epithelial permeability, resistance to metronidazole, and enteroadhesion in a human colonic cell line [49]. It also has been determined that ST3 exhibits extreme intra-ST diversity [50], whereas ST4 genomes appear fairly conserved [50], and this could explain why the two strains of ST4 (WR1 and WR2) do not produce significantly distinct effects on *B. vulgatus* (in terms of growth, modification of TEER, permeability, and gene expression).

## Conclusion

This is the first study to show the interactions between *Blastocystis* ST4 and several intestinal bacteria in vitro. We observed higher bacterial CFU counts when gut commensal bacteria were co-incubated with *Blastocystis* ST4, while the growth of *B. vulgatus* was inhibited. These results indicate that *Blastocystis* ST4 can interact with gut commensal bacteria, and lead to differential changes in the gut microbiota. Furthermore, co-incubation with *Blastocystis* ST4 was able to maintain the epithelial barrier in HT-29 monolayers, and ameliorate *B. vulgatus*-mediated barrier damage.

## Supplementary Information

**Additional file 1: Table S1.** qPCR primers used in this study.

## Data Availability

The datasets generated during and/or analyzed during the current study are available from the corresponding author on reasonable request.

## Abbreviations

qPCR: Quantitative polymerase chain reaction
ROS: Reactive oxygen species
TEER: Transepithelial electrical resistance
SSU rRNA: Small subunit ribosomal RNA
rCDI: Recurrent *Clostridioides difficile* infections
FMT: Fecal microbiota transplantation
FGFP: Flemish Gut Flora Project
AGP: American Gut Project
NUS: National University of Singapore
IMDM: Iscove’s modified Dulbecco’s medium
ATCC: American Type Culture Collection
BHI: Brain heart infusion
CFU: Colony-forming unit
PBS: Phosphate-buffered saline
HBSS: Hank’s balanced salt solution
LPS: Lipopolysaccharide

## References

[1] Andersen LO, Stensvold CR (2016). *Blastocystis* in health and disease: are we moving from a clinical to a public health perspective?. J Clin Microbiol.

[2] Clark CG, van der Giezen M, Alfellani MA, Stensvold CR (2013). Recent developments in *Blastocystis* research. Adv Parasitol.

[3] Yason JA, Liang YR, Png CW, Zhang Y, Tan KSW (2019). Interactions between a pathogenic *Blastocystis* subtype and gut microbiota: in vitro and in vivo studies. Microbiome.

[4] Céline N, Julien S, Bruno P, Christina NM, Ivan W, Amandine C (2014). *Blastocystis* is associated with decrease of fecal microbiota protective bacteria: comparative analysis between patients with irritable bowel syndrome and control subjects. PLoS ONE.

[5] Audebert  C, Even G , Cian A , Group  BI, Loywick A , Merlin S  (2016). Colonization with the enteric protozoa *Blastocystis* is associated with increased diversity of human gut bacterial microbiota. Sci Rep.

[6] Tito RY, Chaffron S, Caenepeel C, Lima-Mendez G, Wang J, Vieira-Silva S (2019). Population-level analysis of *Blastocystis* subtype prevalence and variation in the human gut microbiota. Gut.

[7] Deng L, Wojciech L, Gascoigne NRJ, Peng G, Tan KSW (2021). New insights into the interactions between *Blastocystis*, the gut microbiota, and host immunity. PLoS Pathog.

[8] Maloney JG, da Cunha MJR, Molokin A, Cury MC, Santin M (2021). Next-generation sequencing reveals wide genetic diversity of *Blastocystis* subtypes in chickens including potentially zoonotic subtypes. Parasitol Res.

[9] Stensvold CR, Tan KSW, Clark CG (2020). Blastocystis. Trends Parasitol.

[10] Beghini F, Pasolli E, Truong TD, Putignani L, Cacciò SM, Segata N (2017). Large-scale comparative metagenomics of *Blastocystis*, a common member of the human gut microbiome. ISME J.

[11] Alfellani MA, Stensvold CR, Vidal-Lapiedra A, Onuoha ESU, Fagbenro-Beyioku AF, Clark CG (2013). Variable geographic distribution of *Blastocystis* subtypes and its potential implications. Acta Trop.

[12] Forsell J, Bengtsson-Palme J, Angelin M, Johansson A, Evengård B, Granlund M (2017). The relation between *Blastocystis* and the intestinal microbiota in Swedish travellers. BMC Microbiol.

[13] Nourrisson C, Scanzi J, Pereira B, NkoudMongo C, Wawrzyniak I, Cian A (2014). *Blastocystis* is associated with decrease of fecal microbiota protective bacteria: comparative analysis between patients with irritable bowel syndrome and control subjects. PLoS ONE.

[14] Terveer EM, van Gool T, Ooijevaar RE, Sanders I, Boeije-Koppenol E, Keller JJ (2020). Human transmission of *Blastocystis* by fecal microbiota transplantation without development of gastrointestinal symptoms in recipients. Clin Infect Dis.

[15] Goodrich JK, Waters JL, Poole AC, Sutter JL, Koren O, Blekhman R (2014). Human genetics shape the gut microbiome. Cell.

[16] McDonald D, Hyde E, Debelius JW, Morton JT, Gonzalez A, Ackermann G (2018). American Gut: an open platform for citizen science microbiome research. mSystems..

[17] Chen XQ, Singh M, Ho LC, Moe KT, Tan SW, Yap EH (1997). A survey of *Blastocystis* sp. in rodents. Lab Anim Sci.

[18] Zweibaum A, Laburthe M, Grasset E, Louvard D. Use of cultured cell lines in studies of intestinal cell differentiation and function. Compr Physiol. p. 223–55.

[19] Ray PD, Huang BW, Tsuji Y (2012). Reactive oxygen species (ROS) homeostasis and redox regulation in cellular signaling. Cell Signal.

[20] Weiss GA, Hennet T (2017). Mechanisms and consequences of intestinal dysbiosis. Cell Mol Life Sci.

[21] Kostic AD, Xavier RJ, Gevers D (2014). The microbiome in inflammatory bowel disease: current status and the future ahead. Gastroenterology.

[22] Wu Z, Mirza H, Teo JD, Tan KS (2014). Strain-dependent induction of human enterocyte apoptosis by *Blastocystis* disrupts epithelial barrier and ZO-1 organization in a caspase 3- and 9-dependent manner. BioMed Res Intern..

[23] Deng L, Tay H, Peng G, Lee JWJ, Tan KSW (2021). Prevalence and molecular subtyping of *Blastocystis* in patients with *Clostridium difficile* infection. Singapore Parasi Vect.

[24] Gabrielli S, Furzi F, Fontanelli Sulekova L, Taliani G, Mattiucci S (2020). Occurrence of *Blastocystis*-subtypes in patients from Italy revealed association of ST3 with a healthy gut microbiota. Parasite Epidemiol Control..

[25] Lepczyńska M, Dzika E (2019). The influence of probiotic bacteria and human gut microorganisms causing opportunistic infections on *Blastocystis* ST3. Gut Pathog.

[26] Holt JF, Kiedrowski MR, Frank KL, Du J, Guan C, Broderick NA (2015). *Enterococcus faecalis* 6-phosphogluconolactonase is required for both commensal and pathogenic interactions with *Manduca sexta*. Infect Immun.

[27] Hong HA, Khaneja R, Tam NM, Cazzato A, Tan S, Urdaci M (2009). *Bacillus subtilis* isolated from the human gastrointestinal tract. Res Microbiol.

[28] Leimbach A, Hacker J, Dobrindt U (2013). *E. coli* as an all-rounder: the thin line between commensalism and pathogenicity. Curr Top Microbiol Immunol.

[29] Schell MA, Karmirantzou M, Snel B, Vilanova D, Berger B, Pessi G (2002). The genome sequence of *Bifidobacterium longum* reflects its adaptation to the human gastrointestinal tract. Proc Natl Acad Sci USA.

[30] Plaza-Díaz J, Ruiz-Ojeda FJ, Vilchez-Padial LM, Gil A (2017). Evidence of the anti-inflammatory effects of probiotics and synbiotics in intestinal chronic diseases. Nutrients.

[31] Sales-Campos H, Soares SC, Oliveira CJF (2019). An introduction of the role of probiotics in human infections and autoimmune diseases. Crit Rev Microbiol.

[32] Lim S-M, Jeong J-J, Jang S-E, Han MJ, Kim D-H (2016). A mixture of the probiotic strains *Bifidobacterium longum* CH57 and *Lactobacillus brevis* CH23 ameliorates colitis in mice by inhibiting macrophage activation and restoring the Th17/Treg balance. J Funct Food.

[33] Kelly D, Conway S, Aminov R (2005). Commensal gut bacteria: mechanisms of immune modulation. Trends Immunol.

[34] Maskell JP (1981). The pathogenicity of *Bacteroides fragilis* and related species estimated by intracutaneous infection in the guinea-pig. J Med Microbiol.

[35] Ramanan D, Tang MS, Bowcutt R, Loke P, Cadwell K (2014). Bacterial sensor Nod2 prevents inflammation of the small intestine by restricting the expansion of the commensal *Bacteroides vulgatus*. Immunity.

[36] Bloom SM, Bijanki VN, Nava GM, Sun L, Malvin NP, Donermeyer DL (2011). Commensal *Bacteroides* species induce colitis in host-genotype-specific fashion in a mouse model of inflammatory bowel disease. Cell Host Microb.

[37] Bamba T, Matsuda H, Endo M, Fujiyama Y (1995). The pathogenic role of *Bacteroides vulgatus* in patients with ulcerative colitis. J Gastroenterol.

[38] Paiva CN, Bozza MT (2014). Are reactive oxygen species always detrimental to pathogens?. Antioxid Redox Signal.

[39] O'Rourke EJ, Chevalier C, Pinto AV, Thiberge JM, Ielpi L, Labigne A (2003). Pathogen DNA as target for host-generated oxidative stress: role for repair of bacterial DNA damage in Helicobacter pylori colonization. Proc Natl Acad Sci USA.

[40] Praticò D (2001). In vivo measurement of the redox state. Lipids.

[41] Turner JR (2009). Intestinal mucosal barrier function in health and disease. Nat Rev Immunol.

[42] Ruseler-van Embden JG, van der Helm R, van Lieshout LM (1989). Degradation of intestinal glycoproteins by *Bacteroides vulgatus*. FEMS Microbiol Lett.

[43] Derrien M, van Passel MW, van de Bovenkamp JH, Schipper RG, de Vos WM, Dekker J (2010). Mucin-bacterial interactions in the human oral cavity and digestive tract. Gut Microb.

[44] Huang YL, Chassard C, Hausmann M, von Itzstein M, Hennet T (2015). Sialic acid catabolism drives intestinal inflammation and microbial dysbiosis in mice. Nature Comm.

[45] Ohkusa T, Yoshida T, Sato N, Watanabe S, Tajiri H, Okayasu I (2009). Commensal bacteria can enter colonic epithelial cells and induce proinflammatory cytokine secretion: a possible pathogenic mechanism of ulcerative colitis. J Med Microbiol.

[46] Hiippala K, Kainulainen V, Suutarinen M, Heini T, Bowers JR, Jasso-Selles D (2020). Isolation of anti-inflammatory and epithelium reinforcing bacteroides and *Parabacteroides* spp. from a healthy fecal donor. Nutrients.

[47] Giron LB, Tanes CE, Schleimann MH, Engen PA, Mattei LM, Anzurez A (2020). Sialylation and fucosylation modulate inflammasome-activating eIF2 Signaling and microbial translocation during HIV infection. Mucosal Immunol.

[48] Shiba T, Aiba Y, Ishikawa H, Ushiyama A, Takagi A, Mine T (2003). The suppressive effect of bifidobacteria on *Bacteroides vulgatus*, a putative pathogenic microbe in inflammatory bowel disease. Microbiol Immunol.

[49] Wu Z, Mirza H, Tan KS (2014). Intra-subtype variation in enteroadhesion accounts for differences in epithelial barrier disruption and is associated with metronidazole resistance in *Blastocystis* subtype-7. PLoS Negl Trop Dis.

[50] Stensvold CR, Alfellani M, Clark CG (2012). Levels of genetic diversity vary dramatically between *Blastocystis* subtypes. Infect Genet Evol.

