# Supplementary material for: Interactions between Blastocystis subtype ST4 and gut microbiota in vitro
Source: Parasit Vectors. 2022 Mar 8;15:80. doi: 10.1186/s13071-022-05194-x (PMC8902775; doi:10.1186/s13071-022-05194-x)
Supplement: Supplementary file 1 — Additional file 1: Table S1. qPCR primers used in this study. [file 13071_2022_5194_MOESM1_ESM.docx]

Table S1. Primers used for *B. vulgatus* qRT-PCR

| Gene (ID) | Protein function | Primer sequence (5’-3’) | | Product length |
| --- | --- | --- | --- | --- |
|  |  | Forward | Reverse |  |
| trxB (5304587) | thioredoxin-disulfide reductase | CGTAGCTGTAGTAGGTGGCG | CTTGGATGCGCGTAAGAACG | 112 |
| trxA (5303333) | thioredoxin | GTCGCCACAATTGCTTACCG | GGACCTTGCAAAGCCATGAA | 172 |
| ahpC (5302891) | peroxiredoxin | CATTACGCCCGATGCTGTTG | GCGTGCATTTGGTGTGATGA | 119 |
| ahpF (5301813) | alkyl hydroperoxide reductase subunit F | CAACCCTTTACGAGCCGAGT | AGAAGCTGGAAGCCCGTTAC | 128 |
| BVU_RS16335 (5304208) | ferredoxin | CTGCGTGTCTCGCAAAGAAG | AGAACATGACCCGTGTGTCC | 119 |
| gap (5304545)  House keep gene | type I glyceraldehyde-3-phosphate dehydrogenase | TACGCCAGCCAATTCACCTT | GCGTGTTCCGACTTTGGATG | 121 |
